# Longitudinal profile of iron accumulation in good‐grade subarachnoid hemorrhage

**DOI:** 10.1002/acn3.341

**Published:** 2016-09-01

**Authors:** Christoph Scherfler, Alois Josef Schiefecker, Margarete Delazer, Ronny Beer, Thomas Bodner, Georg Spinka, Mario Kofler, Bettina Pfausler, Christian Kremser, Michael Schocke, Thomas Benke, Elke R. Gizewski, Erich Schmutzhard, Raimund Helbok

**Affiliations:** ^1^ Department of NeurologyMedical University of InnsbruckInnsbruckAustria; ^2^ Neuroimaging Research Core FacilityMedical University of InnsbruckInnsbruckAustria; ^3^ Department of RadiologyMedical University of InnsbruckInnsbruckAustria; ^4^ Department of NeuroradiologyMedical University of InnsbruckAnichstrasse 35A‐6020InnsbruckAustria

## Abstract

**Objective:**

MRI parameters of iron concentration (R2*, transverse relaxation rate), microstructural integrity (mean diffusivity and fractional anisotropy), as well as gray and white matter volumes were analyzed in patients with subarachnoid hemorrhage (SAH) and uncomplicated clinical course to detect the evolution of brain tissue changes 3 weeks and 12 months after ictus.

**Methods:**

MRI scans of 14 SAH patients (aneurysm of the anterior communicating artery, *n* = 5; no aneurysm *n* = 9) were compared with 14 age‐matched healthy control subjects. Statistical parametric mapping (SPM) was applied to objectively identify focal changes of MRI parameters throughout the entire brain and to correlate image parameters with neuropsychological measures.

**Results:**

SPM localized significant bilateral increases in R2* signal within the white matter compartment of the temporal and parietal lobe and the cingulate gyrus (*P* < 0.001) which did not change significantly at 12 months. Significant gray matter volume reduction of the left insula and superior temporal gyrus (*P* < 0.001), as well as decreases in fractional anisotropy of the cingulate gyrus (*P* < 0.01) were also evident at 12 months. Significant correlations were found between fractional anisotropy signal alterations adjacent to the left middle and superior frontal gyrus and cognitive parameters of executive dysfunction (*P* < 0.001).

**Interpretation:**

The study indicates that iron is trapped predominantly throughout large portions of the white matter compartment in SAH patients at 12 months postbleeding. Increased disintegration of fiber tracts colocalizing with iron overload and correlating with lower executive function performance suggests that the white matter compartment is primarily susceptible toward long‐term damage in patients with good clinical grade SAH.

## Introduction

Despite advances in emergency and neurocritical care management, aneurysmal subarachnoid hemorrhage (SAH) is still a devastating disease with up to 95% of survivors reporting cognitive impairment and poor quality of life.^1^ Factors associated with unfavorable cognitive outcome include initial disease severity, the amount of subarachnoid blood, aneurysm location, and the occurrence of secondary complications such as delayed cerebral ischemia, and elevated intracranial pressure.^2^, ^3^ Cognitive and psychosocial long‐term deficits were also reported in SAH patients with “good clinical grade,” uneventful clinical course, and no evidence of structural brain damage.^4^, ^5^, ^6^, ^7^

Recent advances in novel MRI sequences such as diffusion tensor imaging (DTI) and magnetic resonance relaxometry and subsequent automated image analysis provide detailed information about the microstructural integrity of brain tissue, iron deposition, and changes in gray and white matter volume in the cubic millimeter scale, which remain elusive from the investigator‐driven analysis. However, the application of such voxel‐based techniques to cerebral MRI of SAH patients has been limited by the interference of frequently occurring and in their dimension heterogeneous, structural brain lesions and intervention‐related image artifacts. To the best of our knowledge, only one single cross‐sectional study reported voxel‐based morphometry changes in the gray matter of mainly frontobasal cortical areas in patients with ruptured anterior cerebral artery aneurysms, by masking out MRI signal artifacts.^8^ To overcome analysis‐interfering lesions arising from either intervention‐related artifacts or SAH‐related macroscopic brain lesions, we focused this study on patients with spontaneous nontraumatic SAH without visually detectable structural damage on conventional structural MRI. Parameters of iron deposition, microstructural brain tissue integrity, and gray and white matter volumes were measured simultaneously 3 weeks and 12 months following the bleeding. We aimed to determine (1) the MRI signal alterations of DTI, relaxometry, and gray and white matter volumes at 3 weeks and 12 months after bleeding; (2) the evolution of the signal changes within a year follow‐up; and (3) correlations of signal alterations at the 1‐year follow‐up time point with neuropsychological test performance covering parameters of verbal and visual memory, constructive abilities, as well as executive functions.

## Methods

### Participants

Fourteen patients with spontaneous SAH admitted to the Neurological Intensive Care Unit at the Department of Neurology, Medical University of Innsbruck, were identified from a prospectively established and ongoing in‐house database of 109 consecutively recruited SAH patients. To be eligible, patients had to fulfill the following inclusion criteria: (1) SAH onset ≤24 h before admission, (2) completed follow‐up after 12 months including MRI and neuropsychological testing, and (3) no visually detectable structural lesions or placement of external ventricular drainage on the 3 weeks' and 12 months' structural MRI or CT (Table 1). Of 109 consecutive SAH patients, 62 patients had structural lesions on MRI. From the remaining patients, four underwent ventriculoperioneal shunt placement and nine had clip artifacts not compatible with the image analysis. Twenty patients dropped out because of postoperative transfer to other ICUs (*n* = 5), death within 7 days of SAH onset (*n* = 5), transfer to other countries (*n* = 6), death following non‐neurological diseases (*n* = 3), and decline to further study participation (*n* = 1), rendering 14 patients eligible for the analysis.

**Table 1:** Baseline characteristics, complications, and outcome

		*N* = 14 (%)
Clinical characteristics	
Age (SAH), years (mean, SD)		46.1 ± 12 Range: 20–63
Age (healthy controls), years (mean, SD)		47.1 ± 12 Range: 20–64
Gender (SAH), female		8 (57)
Gender (healthy controls), female		8 (57)
Admission H&H grade	1	9 (64)
	2	4 (29)
	3	1 (7)
Loss of consciousness		0 (0)
Admission APACHE II score		4 (2–5)
Admission radiological characteristics		
mFisher scale	0	1 (7)
	1	1 (7)
	2	1 (7)
	3	6 (43)
	4	5 (36)
SAH sum score		13 Range: 10–15
IVH sum score		0 (0–2)
Aneurysm location AcomA = anterior communicating artery		5 (36)
No aneurysm detected		9 (64)
Aneurysm size above 10 mm		0 (0)
Generalized cerebral edema		0 (0)
Intracerebral hematoma		0 (0)
Surgical procedures		
Hydrocephalus requiring EVD/Shunt		0 (0)
Coiling		5 (36)
Clipping		0 (0)
No aneurysm		9 (64)
Hemicraniectomy		0 (0)
Complications		
Pneumonia		0 (0)
Delayed cerebral infarction		0 (0)
Anemia requiring transfusion		0 (0)
Aneurysm rebleeding		0 (0)
Hyperosmolar therapy		0 (0)
Outcome characteristics		
Length of hospital stay, days		19.5 Range: 17–21
Discharge mRS	0	9 (64)
	1	5 (36)
3‐month mRS	0	9 (64)
	1	5 (36)
12‐months mRS	0	11 (79)
	1	3 (21)

APACHE, acute physiology and chronic health evaluation; EVD, extraventricular drainage; H&H, Hunt&Hess; ICH, intracerebral hemorrhage; mFisher, modified Fisher; mRS, modified Rankin Scale; SAH, subarachnoid hemorrhage.

Fourteen age‐ and gender‐matched healthy individuals (females *n* = 8; mean age: 47.1 years, SD ± 12), with no history of severe head trauma, diabetes, or hypertension selected from the MRI database at the Department of Neurology served as control group. Participants with white matter lesions, hemosiderin deposits, vascular or space‐occupying lesions within the cerebrum or motion artifacts were excluded. The study was approved by the Ethics Committee of the Medical University of Innsbruck. Subjects' written informed consent was obtained according to the Declaration of Helsinki. The clinical care of SAH patients conformed to guidelines set forth by the Neurocritical Care Society, the American Heart Association/American Stroke Association and the European Stroke Organization.^9^, ^10^, ^11^ All patients were followed with every‐other‐day transcranial Doppler sonography (DWL Doppler‐Box system; Compumedics, Singen Germany) and received oral or intravenous nimodipine. Delayed cerebral infarction was defined as appearance of new infarction on CT that was judged to be attributable to cerebral vasospasm by an independent radiologist.

### Neuropsychological assessment

At 12 months follow‐up, participants performed a screening of cognitive functions (MMSE, Mini‐Mental State Examination) and a questionnaire assessing symptoms of depression and anxiety (HADS‐D, Hospital Anxiety and Depression Scale; Hermann et al.; Table 2).^12^, ^13^ Furthermore participants completed a battery of standardized neuropsychological tasks assessing verbal and visual memory, constructive abilities, as well as executive functions. Verbal learning and memory was evaluated by a word list learning task (VLMT, Verbal Learning and Memory Test), including five learning trials (list of 15 unrelated words), a distractor list, immediate recall of the learning list, delayed recall of the learning list, and recognition of the studied items.^14^ The Rey–Osterrieth complex figure test (RCFT) was used for assessing visuoconstructive abilities (copying of a complex figure) and visual memory (recall of the figure form memory after a short delay).^15^ Standardized tests assessed several executive functions, including semantic verbal fluency (animals produced per 60 sec), verbal working memory (WMS‐R, digit span backwards), visual conceptualization (clock drawing), as well as cognitive flexibility and set‐shifting (Trail Making Test).^16^, ^17^, ^18^, ^19^ The Frontal Assessment Battery (FAB) was given as a global screening instrument of executive functions.^20^, ^21^ The FAB is composed of six subtests: conceptualization (find similarities between objects), phonemic fluency (S‐words produced per 60 sec), motor programming (fist–palm–edge motor series), sensitivity to interference (conflicting instructions requiring opposite response to a signal), inhibitory control (go–no go paradigm), and environmental autonomy (suppression of manual grasping behavior). Test duration was approximately 60 min. Patients performed the tests in a single session.

**Table 2:** Medians, interquartile ranges, and frequency (*n*/*N*) of impairments on neuropsychological measures for the patient group

	Median	Percentile 25	Percentile 75	*n*/*N*
MMSE	29	28	30	0/13
Anxiety (HADS‐D)	5	2	8	0/13
Depression (HADS‐D)	1	1	4	0/13
Verbal memory, learning (VLMT)	50	42	56	0/13
Verbal memory, short delay recall (VLMT)	11	7	12	3/13
Verbal memory, long delay recall (VLMT)	10	9	11	2/13
Copying (RCFT test)	33	32	35	4/13
Visual memory recall (RCFT test)	20	19	23	1/13
Clock drawing (CLOX)	13	13	14	0/13
FAB score	17	17	18	0/13
Digit span backwards, test score (WMS‐R)	6	5	6	3/13
Semantic verbal fluency (RWT)	27	22	33	0/13
Trail Making Test A (sec; TMT‐A)	23	21	33	0/13
Trail Making Test B (sec; TMT‐B)	63	55	97	0/13
Quotient TMT‐B/TMT‐A	2.7	2.1	3.2	–

In each test performance was classified as impaired when the patient scored equal or below the 5th percentile of age scaled norms, or below cut‐off (MMSE, CLOX). MMSE performance was classified as impaired when the score was below 27, for the CLOX task when below 11. Anxiety and depression were scored as increased when higher than 10.

CLOX, Clock Drawing FAB, Frontal Assessment Battery; HADS‐D, Hospital Anxiety Depression Scale; MMSE, Mini‐mental state; RCFT, Rey Complex Figure Test; RWT, Regensburger Verbal Fluency Test; TMT, Trail Making Test; VLMT, Verbal Learning and Memory Test; WMS‐R, Wechsler Memory Scale‐Revised.

### MRI data acquisition

MRI measurements were performed on a 3‐Tesla whole‐body MR scanner (Magnetom Verio, Siemens Erlangen, Germany) using a 12‐channel head coil at the Department of Neuroradiology at the Medical University of Innsbruck. All participants underwent the same MRI protocol, including whole‐brain T1‐weighted, DTI, and fluid‐attenuated inversion recovery, T2‐ and proton density‐weighted, as well as T2*‐weighted sequences. MRI parameters for coronal T1‐weighted 3D magnetization prepared rapid gradient echo were (magnetization prepared rapid gradient echo) repetition time (TR) = 1800 msec; echo time (TE) = 2.18 msec; inversion time, TI = 900 msec; slice thickness: 1.2 mm; matrix: 256 × 204; number of excitations: 1; flip angel = 9°; field of view 220 mm × 165 mm. DTI data were acquired using single shot spin‐echo echo‐planar imaging (TE/TR = 83/8200 msec, bandwidth = 1596 Hz/pixel; matrix size 116 × 116; 45 axial slices, voxel size 2 × 2 × 3 mm) with 20 diffusion gradient directions with a b‐value of 1000 mm^2^/sec and one reference image with b = 0. For R2* (transverse relaxation rate) quantification a transversal 2D multislice, multiecho gradient echo sequence was used covering the whole‐brain volume: (TR = 200 msec, TE = 2.58 msec, 4.81 msec, 7.04 msec, 9.27 msec, 11.5 msec, 13.73 msec, 15.96 msec and 18.19 msec; flip angle: 20°; bandwidth = 810 Hz/pixel; matrix size 128 × 128; 43 axial slices; voxel size of 1.7 × 1.7 × 3.0 mm). R2* maps were calculated by pixel‐wise fitting of a monoexponential model to the signal values of the respective gradient echoes. The total duration of the imaging protocol was 27 min 40 sec.

### Image postprocessing

To avoid a priori assumptions through region of interest analysis on brain areas of potential interests gray and white matter volumes, MD, FA, and R2* measures were subjected to statistical parametric mapping (SPM, Wellcome Department of Cognitive Neurology, London, UK), a technique that objectively localizes focal changes of voxel values throughout the entire brain volume.^22^ The software package SPM8 implemented in Matlab 7.8 (Mathsworks Inc., Sherborn, MA) was used to preprocess and analyze MRI data. Voxel‐based morphometry of the gray and white matter compartments was achieved by the standard version of the diffeomorphic anatomical registration using exponentiated lie algebra toolbox (DARTEL) implemented in SPM8 to have a high‐dimensional normalization protocol.^23^ Segmented and modulated images were transformed from group‐specific diffeomorphic anatomical registration into Montreal Neurological Institute (MNI) space and smoothed by a Gaussian kernel of 8 × 8 × 8 mm. To achieve accurate spatial normalization for MD, FA, and R2* images, previously coregistered T1‐weighted images were normalized onto the T1 template in MNI space, and the resulting transformation parameters were applied to the participant's corresponding MD, FA, and R2* images. A Gaussian kernel of 8 × 8 × 8 mm was then convolved with the spatially normalized parametric images to smooth them in order to accommodate interindividual anatomic variability and to improve signal to noise ratios for the statistical analysis. A masking threshold of 10% of the lower image signal was applied to reduce signal noise. For MD, FA, and R2* analyses, age was included as a covariate. For voxel‐based morphometry analysis, age and total intracranial volume were entered as covariates. MRI acquisitions were processed on a Dell Studio XPS 435 T workstation with 8 cores, each with a 2.93‐GHz Intel 7 processor.

### Statistical analysis

Demographic patient data are presented as frequencies (percentage), and median (range). The binomial test was applied to test for the distribution of gender. The obtained MRI datasets allowed for categorical comparisons of gray matter and white matter segments, as well as MD, FA, and R2* maps in analogous voxel regions. A general linear model was set up to compare image parameters between healthy volunteers and patients with SAH at either baseline or follow‐up time point using a one‐way ANOVA design. Longitudinal SAH group comparisons were achieved by a one‐group (SAH) and two conditions (baseline and follow‐up) design. Voxel‐wise correlation analyses were performed by using the general linear model implemented in SPM. MRI parameters including gray matter and white matter volumes, MD, FA, and R2* values, and the scores of neuropsychological tests were entered into a design matrix. The relationship between MRI voxel values and parameters of cognitive performance were examined with *t*‐contrast. Results from SPM analyses were corrected for multiple comparisons and the height threshold was set to *P* < 0.001 for ANOVA as well as correlation analyses and to *P* < 0.01 for longitudinal within group analysis. Neuropsychological variables included verbal memory (short delay free recall, VLMT), visual memory (recall, RCFT), constructive abilities (copy trial, RCFT), working memory (digit span backward), verbal fluency (animals per 60 sec), summary score in the FAB, as well as cognitive flexibility (quotient TMT‐B/TMT‐A). The quotient TMT‐B/TMT‐A was used as this measure has been reported to minimize the influence of other cognitive variables.^24^

## Results

Baseline characteristics, hospital complications, and outcome data are shown in Table 1. There was no significant difference in age and sex distribution between SAH patients and the control cohort (Table 1).

One patient was excluded from the neuropsychological analysis as her depression score (HADS‐D) was above 10 making it difficult to detect a true effect of SAH. The remaining participants all had an MMSE score ≥27. Table 2 gives the median scores in neuropsychological tasks, the interquartile ranges, and the frequencies of patients scoring in the impaired range according to age‐scaled norms.

### Structural abnormalities at 3 weeks and 12 months after SAH

**Figure 1:**
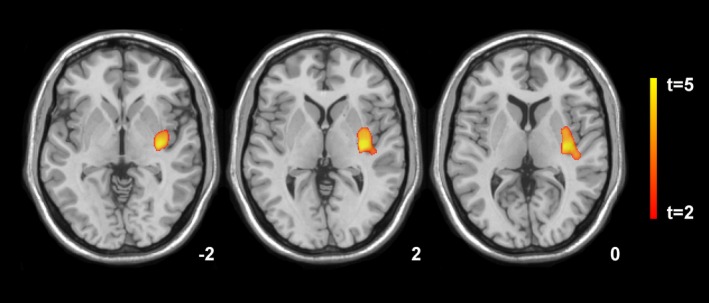
Statistical parametric mapping (*t*) axial intensity projection maps rendered onto a stereotactically normalized MRI scan, showing areas of significant decreases in gray matter volume in a cohort of patients with subarachnoid hemorrhage versus healthy control subjects 12 months after bleeding (color code, yellow to orange). The number at the bottom right corner of each MRI scan corresponds to the *z* coordinate in Montreal Neurological Institute space.

**Figure 2:**
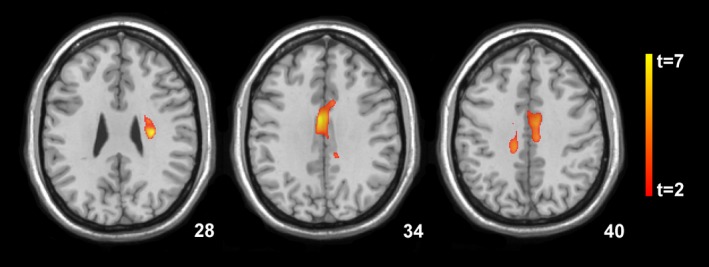
Statistical parametric mapping (*t*) axial maximum intensity projection maps rendered onto a stereotactically normalized MRI scan, showing areas of significant decreases in fractional anisotropy values in a cohort of patients with subarachnoid hemorrhage at 12 months compared to the 3‐week time point (color code, yellow to orange). The number at the bottom right corner of each MRI scan corresponds to the *z* coordinate in Montreal Neurological Institute space.

SPM localized significant decreases in gray matter volume of the left insula, posterior putamen extending to the superior temporal gyrus (Brodmann Area [BA] 48) at 3 weeks following SAH compared with the control group (*P* < 0.001), which did not change significantly at 1‐year follow‐up (Fig. 1). No significant volume changes were observed in the white matter compartment at the first and second time points. Significant increases in MD values in the SAH group versus the control group were identified within the area of the left middle temporal gyrus (BA 20, 21) 3 weeks after SAH (*P* < 0.001). No significant MD changes were detected at 1‐year follow‐up when compared with the control group and the first time point. At both MRI investigations, FA values were not significantly altered when compared to the healthy control group, however, significant FA decreases were evident in the white matter compartment adjacent to the middle portion of the cingulate at 1‐year follow‐up compared to the initial time point (*P* < 0.01, Fig. 2, Table 3).

**Table 3:** Statistical parametric mapping findings, showing the locations of significant changes of gray matter volume, mean diffusivity, and transverse relaxation rate changes in patients with subarachnoid hemorrhage at baseline and 1 year follow‐up investigation within group versus healthy control subjects

Cerebral region	Cluster size (mm^3^)	MNI coordinates			*t* value	*P* value corrected at cluster level	Height threshold
		*x*	*y*	*z*			
Significant gray matter volume decreases in patients with SAH at baseline versus healthy subjects							
Left insula, posterior putamen, and superior temporal gyrus, BA 48	7597	40	−14	6	6.00	0.001	0.001
Significant gray matter volume decreases in patients with SAH at follow‐up versus healthy subjects							
Left insula, posterior putamen, and superior temporal gyrus, BA 48	4421	36	−3	4	5.75	0.013	0.001
Significant increases in mean diffusivity in patients with SAH at baseline versus healthy subjects							
Left middle temporal gyrus, BA 20, 21	2970	53	−25	−9	5.07	0.001	0.001
Left frontal superior gyrus, BA 10	2267	10	49	1	4.43	0.015	0.001
Left anterior cingulate, BA 11		12	38	7	4.40
Significant decreases in fractional anisotropy in patients with SAH at baseline versus follow‐up							
White matter compartment adjacent to the middle portion of the cingulate	5808	3	−4	34	6.7	0.001	0.01
Significant R2* increases in patients with SAH at baseline versus healthy subjects							
Area of the interhemispheric fissure extending to parietal and temporal subarachnoid space and adjacent gray and white matter areas	166249	1	−57	46	6.99	0.001	0.001
Significant R2*increases in patients with SAH at follow‐up versus healthy subjects							
Precuneus and middle portion of the cingulate	110817	−2	−58	46	4.66	0.001	0.001
		−4	−31	51	4.54		
Parietal portion of the left centrum semiovale extending to the temporal lobe		24	−50	47	4.42		
		60	−30	9	4.38		
Parietal portion of the right centrum semiovale	16110	−42	−27	48	4	0.009	
		−22	−24	50	3.8		
Right white matter compartment adjacent to middle temporal gyrus	12194	−45	−49	2	4.2	0.034	

MNI, Montreal Neurological Institute coordinates; R2*, transverse relaxation rate, SAH, subarachnoid hemorrhage.

### Brain iron content at 3 weeks and 12 months after SAH

**Figure 3:**
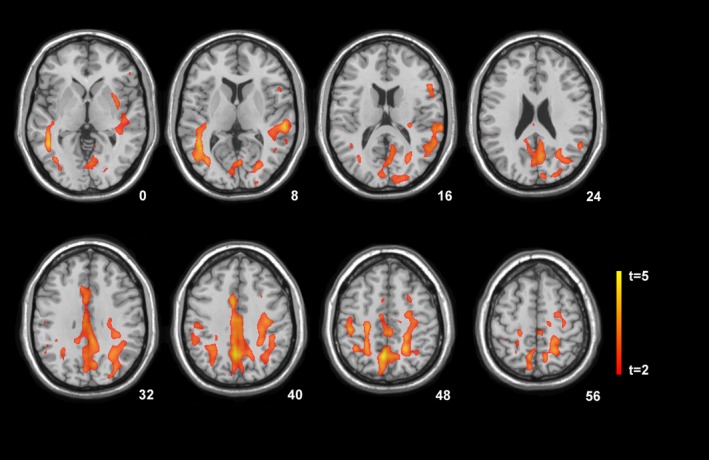
Statistical parametric mapping (*t*) axial intensity projection maps rendered onto a stereotactically normalized MRI scan, showing areas of significant increases in the transverse relaxation rate (R2*) in a cohort of patients with subarachnoid hemorrhage versus healthy control subjects 12 months after bleeding (color code, yellow to orange). The number at the bottom right corner of each MRI scan corresponds to the *z* coordinate in Montreal Neurological Institute space.

SPM localized significant increases in R2* values throughout the entire subarachnoid spaces with highest *t*‐values in the interhemispheric fissure and adjacent brain tissue of the SAH group 3 weeks following bleeding compared with the healthy control group (*P* < 0.001). At 1‐year follow‐up, R2* values were significantly increased in middle portion of the cingulate (bilateral), the parietal portion of the centrum semiovale (bilateral), and the white matter tracts adjacent to the middle temporal gyrus compared to the healthy control cohort (*P* < 0.001, Fig. 3). No significant difference in the R2* signal was detected between the first and second time points (Table 3).

### Correlation between MRI parameters and neuropsychological assessments (Table 4)

**Table 4:** Brain regions of significant positive correlations of fractional anisotropy signal decreases and executive function determined by the Frontal Assessment Battery in patients with subarachnoid hemorrhage at 1‐year follow‐up investigation

Cerebral region	Cluster size (mm^3^)	MNI coordinates			*t* value	*P* value corrected at cluster level	Height threshold
		*x*	*y*	*z*			
White matter tracts adjacent to left frontal superior gyrus, and supplementary motor area, BA 32, 6	484	19	4	47	27.97	0.006	0.001
White matter tracts adjacent to left middle frontal gyrus, BA 44	527	31 29	12 19	37 30	13.06 11.06	0.003	0.001

Age was included as a covariate. Corrected for multiple comparisons. BA, Brodmann area.

**Figure 4:**
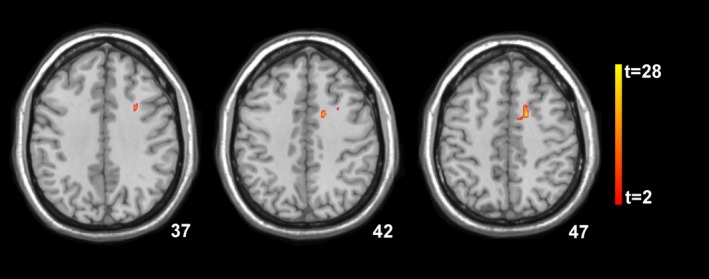
Statistical parametric mapping (*t*) axial intensity projection maps rendered onto a stereotactically normalized MRI scan, showing areas of significant associations correlations between decreased fractional anisotropy and worsening of Frontal Assessment Battery scores in patients with subarachnoid hemorrhage 12 months after bleeding (color code, yellow to orange). The number at the bottom right corner of each MRI scan corresponds to the *z* coordinate in Montreal Neurological Institute space.

Decreases in executive function as determined by the FAB correlated significantly with decreased fractional anisotropy measures of the white matter tracts adjacent to the middle portion of the left cingulate gyrus (*P* < 0.006) and the white matter tracts adjacent to the left middle frontal gyrus of patients with SAH (*P* < 0.003, Fig. 4). No significant associations of regional gray matter and white matter, MD, and R2* changes in patients with SAH were found.

## Discussion

One year after spontaneous nontraumatic SAH, we found substantial iron accumulation in the white and, to a smaller extent, gray matter compartments of the brain with peak depositions within the white matter tracts of the middle portion of the cingulate, precuneus (bilateral) and right middle temporal gyrus. The location of iron accumulation was further associated with microstructural axonal damage. Recently, several studies reported a linear relationship between transverse magnetic resonance relaxation rates and iron content in healthy subjects and postmortem tissue, indicating that R2* imaging is a good surrogate for detecting cerebral iron accumulations.^25^, ^26^, ^27^ By a rater‐driven approach, T2*‐weighted imaging of SAH patients in their chronic stage allowed to identify signal alterations within the subarachnoid space even years after the onset.^28^, ^29^, ^30^ These researchers reported a high predictive value of the T2*‐weighted signal to detect the location of the initial hemorrhage, however low specificity, suggesting diffusion of blood products within the subarachnoid space over time. Histological examination of patients with incidentally detected unruptured intracranial aneurysms showed that hemosiderin was stored in the arachnoid tissue and granulations, indicating this iron‐storage complex to be resistant to washout through cerebrospinal fluid.^31^, ^32^ We are not aware of any studies focusing on whole brain analysis of iron depositions within the brain tissue following SAH using either iron‐sensitive MRI sequences or postmortem histological investigations. By applying SPM to 3D maps of the effective transverse relaxation rate R2* (R2* = 1/T2*), signal alterations strongly suggestive of iron deposition were clearly localized to the gray and white matter compartments of the brain at 1‐year follow‐up which could not have been predicted by visual inspection (Fig. 3). Due to limitations regarding spatial resolution of T2* sequences and signal filtering required for statistical analysis, the proportion of iron storage within the arachnoid matter and the adjacent brain tissue cannot be precisely calculated. However, R2* signal alterations were observed several millimeters distant to the border of the arachnoid mater clearly indicating the storage of iron within brain tissue. Our observation is consistent with animal data showing that hemoglobin released through bleeding into the subarachnoid space penetrates easily into the deeper layers of the cortex and is taken up primarily by microglia following the bleeding.^33^, ^34^ The same authors found that subsequently heme‐oxygenase 1, an enzyme, degrading hemoglobin into carbon monoxide, biliverdin, and iron was markedly increased. Intracellular iron overload was shown to result in lipid peroxidation and free radical formation leading to delayed edema and acute brain injury.^35^, ^36^, ^37^, ^38^, ^39^ In accordance with these findings, brain tissue damage was reported to be reduced in experimental SAH by the knockout of lipocalin 2, an iron transport protein.^40^ In our study, the location of iron deposition was associated with microstructural disintegration of the white matter tracts adjacent to the mid‐cingulate gyrus at 12 months follow‐up supporting the hypothesis of iron‐induced axonal injury, occurring also remotely from the ruptured artery. A more multifocal rather than focal white matter damage was recently identified by DTI and histological markers of membrane disruption and cytoskeletal injury in an animal model of SAH.^41^ Further to this finding and supporting our hypothesis, tau protein, a microtubule‐associated protein usually restricted to brain intracellular compartment, was found in the brain extracellular compartment of the vascular territory of the aneurysm bearing artery in patients with SAH using cerebral microdialysis.^42^

In the current study, we found that axonal damage adjacent to the left middle and superior frontal gyrus and the left supplementary area was associated with the FAB summary score. The FAB provides a useful screening instrument for executive dysfunction as shown in several clinical investigations, including studies on Parkinson's disease, Alzheimer's disease, or stroke.^20^, ^43^, ^44^ Brain imaging studies revealed positive correlations of decreased FAB scores indicating executive dysfunction and reductions of brain tissue perfusion located to the left middle frontal gyrus and the right superior frontal gyrus in patients with MCI, Alzheimer's disease, and frontotemporal lobe dementias.^45^, ^46^, ^47^ The association between lower scores in executive function tasks and frontal white matter microstructural abnormalities found in our study is consistent with this body of evidence. However, it should be noted that the FAB performance of our patient group was within the range of standardized norms and that no association between R2* signal alterations and cognitive functions were found at 1‐year follow‐up. Although it is tempting to link iron overload in those structures with MRI parameters of axonal and neuronal damage as well as measures of cognitive function, other causes such as neuroinflammation, microthrombosis, mitochondrial and endothelial dysfunction, and cortical spreading depolarizations may have also contributed to microstructural brain tissue damage.^48^ Interestingly, none of our patients had clinical or radiographic evidence of cerebral infarctions suggesting a minor role of marked delayed cerebral ischemia in this process. In this context, markers of neuroinflammation would be of great interest as animal data suggested an association between erythrocyte lysis, iron overload, oxidative stress, and brain edema.^37^

One limitation of this study was the selected inclusion of SAH patients without visually detectable structural brain lesions on MRI, which was necessary to eliminate confounders potentially interfering with the automated whole brain analysis. This stratification procedure was inevitably associated with the inclusion of patients with (1) clinically favorable grades and (2) no aneurysm detection in 64% of cases, limiting the generalizability of the results toward a broader SAH spectrum. However, we would like to emphasize that MRI signal alterations indicative of increased iron deposition and microstructural disintegrity were identified even in patients with good clinical grade SAH. The aneurysm location of the remaining 36% of aneursym‐positive SAH patients was located solely to the anterior communicating artery. Therefore, although unlikely, our results may not be extrapolated to SAH patients with aneurysms in other vessel territories.

This study provides the first evidence that iron is trapped predominantly throughout large portions of the white matter compartment in SAH patients with uncomplicated course at 12 months postbleeding. Increases in iron storage were colocalized with microstructural abnormalities of the fiber tracts adjacent to the mid‐cingulate suggesting iron overload as a potential cofactor that might induce prolonged neuronal damage in SAH patients. Further studies are warranted to determine whether the susceptibility of neuronal projections toward long‐term functional and structural deteriorations can be modified by pharmacological intervention targeting SAH‐related intracellular iron accumulation.

## Author Contributions

C. S., M. S., and R. H. were responsible for the study concept and design and together with R. B., T. Be., M. D., R. P., E. G., and E. S. for data interpretation. A. S., T. Bo., and M. K. acquired the clinical data. G. S., C. K., M. S., and E. G. were responsible for acquisition and processing of MRI data. G. S. and C. S. did the analysis of MRI data. C. S. produced a draft of the report, which was reviewed and revised by all other authors. R. H., E. S., and R. B. obtained funding.

## Conflicts of Interest

None declared.
